# Evaluating a personalized treatment for substance use disorder in people with mild intellectual disability or borderline intellectual functioning: A study protocol of a multiple baseline across individuals design

**DOI:** 10.1016/j.conctc.2020.100616

**Published:** 2020-07-16

**Authors:** Lotte C.F. Gosens, Roy Otten, Robert Didden, Evelien A.P. Poelen

**Affiliations:** a Research and Development, Pluryn, P.O. Box 53, 6500, AB, Nijmegen, the Netherlands; b Behavioral Science Institute, Radboud University, Nijmegen, P.O. Box 9104, 6500, HE, Nijmegen, the Netherlands; c REACH Institute, Arizona State University, Tempe, USA; d Trajectum, Zwolle, the Netherlands

**Keywords:** Substance use disorder, Mild intellectual disabilities, Borderline intellectual functioning, Personalized treatment, mHealth application

## Abstract

**Background:**

The prevalence of substance use disorder (SUD) in people with Mild Intellectual Disabilities or Borderline Intellectual Functioning (MID-BIF) is high. However, an effective personalized treatment for people with MID-BIF and SUD is lacking. This paper describes the study protocol of the effectiveness study of Take it Personal!+, a personalized treatment for people with MID-BIF and SUD.

**Methods:**

The effectiveness of Take it Personal!+ in decreasing substance use will be assessed in a non-concurrent multiple baseline across individuals design comprising ten participants with MID-BIF and SUD. The participants with MID-BIF and SUD will be randomly allocated to one of the five baseline lengths (7–11 days). Substance use, will be assessed during baseline, intervention, and post-treatment phase using a daily diary method.

**Discussion:**

If this study shows that Take it Personal!+ is effective in decreasing substance use, the gap in treatment for people with MID-BIF and SUD will be filled.

**Trial registration:**

The study is registered in the Netherlands Trial Register (Trial NL4935, registered July 2, 2019).

## 1 Introduction

The prevalence of Substance Use (SU) in people with Mild Intellectual Disability or Borderline Intellectual Functioning (MID-BIF; 50–85) is alarming [1,2]. People with MID-BIF are more vulnerable to problematic substance use (due to e.g., poor refusal skills and impulse control), and the prevalence of Substance Use Disorder (SUD) in this target group is high, particularly in individuals with comorbid psychiatric disorder, individuals receiving residential or outpatient Intellectual Disabilities (ID) care, and forensic patients [3,4]. Besides, individuals with MID-BIF are at increased risk for developing SUD, due to various risk factors (e.g. impairment in cognitive and adaptive skills, lack of adequate coping skills, peer pressure) [4,5]. SUD has severe adverse health and social consequences for people with MID-BIF [6]. SUD is associated with comorbid mental health problems (e.g., depression and anxiety), behavioral problems (e.g., aggression and criminal activities), social problems (e.g., loneliness, conflicts with families and friends), and financial problems [6,7]. In addition, SUD hinders participation and inclusion in society [1].

Despite the urgent need for SUD interventions for people with MID-BIF, effective interventions that meet the needs of these people and appropriately address the level of SUD severity are lacking. Individuals with MID-BIF do not benefit from regular interventions because these interventions are not adjusted to their needs. People with MID-BIF have deficits in intellectual and adaptive functioning (e.g. in the areas of language, memory, organizing tasks, generalization of learned skills) [8], and interventions need to be adjusted to these needs. Over the past years, a range of interventions have been developed for this vulnerable group [4,5,[9], [10], [11], [12]]. Some of these interventions are educational or preventive or aiming to increase motivation or prevent individuals from start using substances [9,11,12]. However, these interventions fall short in individuals who present with more severe levels of SUD [11,12]. Another point is that the effectiveness of current interventions is mostly unknown due to lack of research [4]. Recently, the focus in mental health care shifted towards more personalized treatment approaches. Personalized treatment seems to be necessary in achieving recovery, and it prevents under- or over-treatment [13]. Personalized interventions are personalized based on characteristics of the client [14]. Current interventions for SUD in individuals with MID-BIF have been designed according to a “one-size-fits-all” mindset, which means that the intervention is not protocolled to target specific characteristics. Take it Personal!+ responds to the need for effective personalized treatment for people with MID-BIF and SUD.

### 1.1 Take it Personal!+

Take it Personal!+ is a personalized SUD treatment for people with MID-BIF. The treatment aims to reduce SU. Take it Personal!+ is based on the personality profile (sensation seeking, impulsivity, anxiety-sensitivity, or negative thinking) of the client. Studies have shown that these personality profiles are associated with SU in people with and without MID-BIF [15,16]. Prevention programs based on these personality profiles have shown to be more effective compared to non-personalized prevention [17] and to be effective in decreasing SU in youth with MID-BIF [18]. Preliminary empirical findings suggest that SUD treatment for people with MID-BIF may also benefit from differentiating personality profiles.

Furthermore, Take it Personal!+ is based on Motivational Interviewing (MI) and Cognitive Behavioral Therapy (CBT). MI and CBT are effective methods in decreasing SU in individuals without MID-BIF [19,20] and adapted versions of MI and CBT have shown promising results in increasing motivation and reducing SU in individuals with MID-BIF [9,18,21,22]. In addition, Take it Personal!+ is supported by a mHealth application (TiP!) which supports the transfer-of-treatment to everyday life. Digital support is increasingly being applied in interventions [23,24] and may eliminate problems with generalization and transfer, which are often seen in individuals with MID-BIF.

Take it Personal!+ is adjusted to the needs of individuals with MID-BIF. All communication materials are simplified and supported with pictures, the treatment design with an A and B session ensures repetition and a confidant of the participant is involved during the intervention to stimulate generalization. More information about the adjustments is described in the intervention mapping paper [25].

### 1.2 The study

This study aims to evaluate the effectiveness of Take it Personal!+ in decreasing SU in individuals with MID-BIF and SUD. We hypothesize that Take it Personal!+ will decrease SU in these individuals. If confirmed, Take it Personal!+ will fill the current gap in the effective treatment interventions for SUD in people with MID-BIF.

## 2 Methods

### 2.1 Study design

Data will be collected within a non-concurrent multiple baseline across individuals design [26]. We will examine the effectiveness of Take it Personal!+ in ten participants with MID-BIF and SUD receiving care from healthcare organizations for people with MID-BIF and behavioral problems in the Netherlands. Participants will be randomly allocated to one of the five baseline lengths varying from 7 to 11 days. The onset of the treatment will be randomized, which will maximize internal validity [27]. The design consist of four phases: baseline phase (7–11 days), intervention phase (11 weeks), post-treatment (1 month), and a follow-up phase (3 months after the intervention). The study is conducted between spring 2019 and summer 2020. It is registered in the Netherlands Trial Register (Trial NL4935, registered July 2nd, 2019), and approved by the Faculty Ethics Committee of the Radboud University (ECSW-2019-033).

### 2.2 Recruitment

Clients with MID-BIF and SUD will be recruited from different healthcare organizations for people with MID-BIF and behavioral problems to examine the effectiveness of Take it Personal!+ in ten participants. Professionals can invite clients to participate in the study, or clients can ask professionals for a treatment for their SU problem. After the invitation, clients will be informed about the research and given one week to reflect on and sign the informed consent form. Permission of parents or legal representatives will be asked, if necessary. During the intake procedure, the Take it Personal!+ therapist and a physician (e.g., intellectual disability physician, psychiatrist, general practitioner, addiction physician) will assess whether the client meets the following inclusion criteria: 1) diagnosed with a MID-BIF with an IQ between 60 and 85 as assessed by a standardized intelligence test, and deficits in adaptive skills, according to the 5th edition of the Diagnostic and Statistical Manual of Mental Disorders (DSM-5) [8], 2) diagnosed with a SUD with cannabis, alcohol, XTC, cocaine, or speed use according to the DSM-5 [8], 3) substance use at least three days a week, and 4) proficient in Dutch language. Clients will not be eligible if they meet one of the following exclusion criteria: 1) risk of severe withdrawal symptoms, 2) severe psychiatric comorbidity, such as suicidality, psychosis, or major depressive disorder, 3) severe somatic problems, and 4) psychosocial problems that interfere with treatment (e.g. homelessness). Clients that meet the exclusion criteria will be referred to another treatment or treatment centre (e.g., a client with a high risk of withdrawal symptoms will be referred to addiction care).

### 2.3 Treatment

The aim of Take it Personal!+ is to decrease SU. The treatment is personalized (i.e., adapted to the participant's personality profile related to SUD), based on MI and CBT and supported by an mHealth application TiP! Take it Personal!+ is based on a CBT-MI protocol for SUD in youths [28] and a CBT-MI protocol for SUD in individuals with MID-BIF [29]. In Take it Personal!+ these protocols are personalized and further developed in collaboration with professionals working in the field of addiction and intellectual disability care.

Take it Personal!+ is designed to last for 11 weeks; however, the therapist can adjust the duration based on the needs of the participant (e.g. if a participant needs more repetition). Take it Personal!+ offers two sessions (Session A and B) of 45 min per week. Session A is an individual session with the participant and in session B the participant brings along a confidant from his/her social network or professional care. Therapists with experience in treating individuals with intellectual disabilities and SUD will carry out the treatment which is based on the therapist manual. All therapists are CBT and MI-certified and experienced in ID treatment.

At intake, the Substance Use Risk Profile Scale (SURPS) [30] will be completed to allocate the participant to one of the four personality profiles. The Take it Personal!+ manual is adapted to the personality profiles and consist of the following key components: 1) motivation to change SU, 2) psycho-education regarding the personality profile, 3) setting goals and making a plan to change SU, 4) identification of personality profile and associated signals of problematic behavior, 5) functional analysis of SU, 6) increasing self-control, 7) behavioral and cognitive coping skills training, and 8) relapse prevention.

The treatment is supported by the mHealth application TiP! The application consists of the following components closely related to the key components of the treatment sessions: 1) exercises (e.g. disadvantages of SU and advantages of changing SU), 2) week goals and how to achieve them, 3) future wishes and how to achieve them, 4) a help! button (i.e. personalized feedback on risk situations, craving, and relapse and the possibility to text or call someone from his/her address book), 5) information about self-control skills, refusal skills, and positive and negative thoughts, and 6) a relapse prevention plan. In addition, gamification by using a personalized avatar and rewards in terms of accessories for the avatar and points for personal rewards makes TiP! appealing for users. For more information about the key components of the treatment and mHealth application see the intervention mapping paper [25]. During the treatment, collaboration with addiction care is important, therapists stay in close contact with different professionals who working in addiction care and are available for consultation.

### 2.4 Outcomes

The primary outcome of this study is frequency and quantity of SU. SU will be assessed with daily diary measures during the baseline phase (7–11 days), intervention phase (11 weeks), and post-treatment (1 month). The daily diary method will be applied using a mobile application for cellular phones (Ethica) [31]. Every morning or evening, participants will receive a push notification to complete a short questionnaire within a determined time frame (i.e., varying from 240 min to 780 min). The time of the push notification and the time frame will be decided in consultation with the participant. The questionnaire consists of fifteen questions and is based on previous studies [32,33]. One item will assess the frequency of SU, ‘Did you use *the primary substance* today?’ measured by (1) ‘yes’ and (2) ‘no’ responses. If the participant answers positively, he or she will be asked the second open-ended question, to assess the quantity of SU, ‘How many times?‘. To monitor the frequency of other daily SU, participants will be asked the question, ‘Did you use another substance today?‘, measured by personalized response categories (e.g., (1) ‘no’, (2) ‘yes, alcohol’, (3) ‘yes, XTC’, (4) ‘yes, cocaine’, (5) ‘yes, something else’).

A briefing by the researcher on the daily diary method will take place the day before the start of the baseline phase. In this briefing, attention will be paid to the daily diary procedure, the privacy statement, understanding and personalization of the items, and the time of the daily push notification will be determined. On the second day of the baseline phase, the researcher will visit the participants to check whether the participants have any questions or problems with using the application. If needed, the researcher will plan a second visit later that week [34]. Moreover, during the entire study the researcher will contact the participants if they did not fill in a number of questionnaires. Part of the questions will also be discussed during treatment sessions, where the therapist will evaluate the procedure during the intervention phase.

Additional information on SU will be collected using standardized questionnaires. At baseline, post-treatment, and follow-up, we will use the Substance Use and Misuse in Intellectual Disability - Questionnaire (SumID-Q) [35] to assess the frequency, quantity and severity of SU by the Alcohol Use Disorder Identification Test (AUDIT) [36] and the Drug Use Disorder Identification Test (DUDIT) [37].

To monitor treatment fidelity, therapists will fill out an evaluation form after each session. The form will assess 1) adherence to the manual, 2) dosage, and 3) program differentiation [38].

### 2.5 Statistical analyses

To determine whether Take it Personal!+ leads to a decrease in SU, data on SU gathered from the daily diary measures will be analyzed visually and by using time series analysis. Both within-person processes and between-person differences will be studied. Missing data will be imputed using Kalman filtering equations [39] with the R package imputeTS [40]. At least a medium effect size is expected (cf [18]). The results will be reported following the Single-Case Reporting guideline in Behavioral Interventions (SCRIBE) to make the study transparent, to facilitate replication, and to structure the paper.

## 3 Discussion

Take it Personal!+ has the potential to fill the gap in the SUD treatment for individuals with MID-BIF and SUD. Take it Personal!+ is a personalized treatment that differentiates between SUD-related personality profiles of clients. In the multiple baseline across individuals design we aim to test the effectiveness of Take it Personal!+ in decreasing SU.

### 3.1 Strengths and limitations

The study contains several innovative elements and strengths. First, Take it Personal!+ is the first personalized SUD treatment supported by mHealth application for individuals with MID-BIF. Second, Take it Personal!+ is based on theoretical frameworks (i.e., MI, CBT, and the four personality profiles) that have shown positive results in a range of studies on SUD [9,[17], [18], [19], [20],^22^]. Third, Take it Personal!+ is responsive to the learning style and needs of individuals with MID-BIF. The CBT is adjusted to the needs of individuals with MID-BIF (e.g., simplified communication), and the design of two sessions a week ensures repetition. Moreover, individuals with MID-BIF experience difficulties in the generalization of treatment to everyday life. In Take it Personal!+, both the confidant from the participant's social or professional network and the mHealth application play essential roles in the treatment and increase the transfer-of-treatment to everyday life.

A common criticism of Single-Case Experimental Designs is the lack of generalizability of the results. In the current study the generalizability of the results is increased in different ways. First, we use direct replications (e.g., same therapist) and systematic replications (e.g., different participants’ characteristics, different therapists, and different settings) [38,41]. The extent to which the intervention effect is comparable across different replications will provide information about the generalizability of the results [27,41]. Second, the characteristics of the participants will be described precisely, which will increase the generalizability by the possibility to apply the intervention to clients with similar characteristics. Lastly, social validity will be assessed and reported [41]. Another strength of the study is that the daily diary method will generate many data points. According to the standards of single-case designs, each phase should have at least 3 data points to allow for time series analyses, but ideally, 5 or more data points should be included [42]. In this study, each phase has more than 5 data points. Another strength is the overlap between the treatment and the data collection, which minimizes the burden of the research for participants. The treatment also requires individuals to register SU in a daily diary. The answers to the daily diary questions for the research will be shared with the therapist, who will discuss them in the treatment sessions.

The study also has some limitations. First, most of the outcome data will be based on self-reports (i.e., questionnaires and daily diary measures), which could lead to a measurement error due to response bias. However, most studies on SU use self-reports, and an earlier study showed that the results of the SumID-Q did not differ from biomarker analysis [43]. Another limitation is that as far as we know, this is the first study to apply daily diary measures in people with MID-BIF for a longer period of time. However, the feasibility, validity, and reliability of this method has been tested in adults with intellectual disabilities [44]. In this study participants filled out more questionnaires in one day for a shorter period of time. Based on this study, and the fact that more questionnaires in one day is more burdensome to complete as compared to one measure a day, the expectation is that the daily diary method for a longer period of time is applicable in people with MID-BIF.

## Ethics approval and consent to participate

The study was approved by the Faculty Ethics Committee of the Radboud University (ECSW-2019-033). All participants sign the informed consent form and if necessary parents or legal representatives also sign the informed consent form.

## Funding

This study is funded by The 10.13039/501100001826Netherlands Organisation for Health Research and Development (10.13039/501100001826ZonMw project 636330005).
